# Using On-Farm Monitoring of Ergovaline and Tall Fescue Composition for Horse Pasture Management

**DOI:** 10.3390/toxins13100683

**Published:** 2021-09-25

**Authors:** Krista La Moen Lea, S. Ray Smith

**Affiliations:** Department of Plant and Soil Sciences, University of Kentucky, N-222C Ag. Science Center North, Lexington, KY 40546, USA; Raysmith1@uky.edu

**Keywords:** ergovaline, horse pastures, pregnant mares, species composition

## Abstract

Central Kentucky horse pastures contain significant populations of tall fescue (*Schedonorus arundinacea* (Schreb.) Dumort) infected with an endophyte (*Epichloë coenophialum* (Morgan-Jones and Gams) Bacon and Schardl) known to produce several ergot alkaloids, with ergovaline in the highest concentration. While most classes of horses are not adversely affected by average levels of ergovaline in pastures, late term pregnant mares have a low tolerance to ergovaline and the related ergot alkaloids. Endophyte-infected tall fescue has been known to cause prolonged gestation, thickened placenta, dystocia, agalactia, and foal and mare mortality. The University of Kentucky Horse Pasture Evaluation Program utilizes ergovaline and endophyte testing, as well as pasture species composition, to calculate ergovaline in the total diet in broodmare pastures. This data is used to develop detailed management recommendations for individual pastures. Application of these recommendations has led to reduced tall fescue toxicity symptoms on these farms, as well as improved pasture management and improved forage quality and quantity.

## 1. Introduction

Tall fescue, *Lolium arundinaceum* (Schreb.) Darbysh; *Schedonorus phoenix* (Scop.) Holub, a cool-season, perennial bunch-type grass, dominates over 15 million ha in the southeastern US [1] including approximately 2 million ha in the state of Kentucky. While the grass has good forage quality and palatability, most plants are infected with a common toxic endophyte, *Epichloë coenophialum*. The endophyte and plant form a symbiotic relationship, with the plant provides nutrients and a hospitable environment for the endophyte. The presence of *E. coenophialum* is beneficial to the plant by increasing pest, drought and grazing tolerance [2,3], but is also toxic to grazing livestock. The endemic nature of infected tall fescue in the U.S. traces back to the widespread distribution of the cultivar Kentucky 31+ containing *E. coenophialum* in the 1940s, 1950s, and 1960s [4]. This cultivar continues to be distributed in the U.S. and other countries due to its superior environmental and edaphic adaptation.

A number of ergot alkaloids are present in infected tall fescue, including ergovaline, ergotamine, ergocrystine, and lysergic acid [5]. Ergovaline has been identified as the most prevalent of the ergot alkaloids, representing roughly 80% of total ergot alkaloids produced [6] and is known to be vasoconstrictive, as documented by measurements of the palmar artery and vein [7]. For this reason, ergovaline has historically been the focus of much livestock research and has been correlated with negative physiological reactions in cattle, horses, and small ruminants consuming toxic endophyte-infected tall fescue.

In cattle, reduced average daily gain (ADG) [8], rough hair coat [9], poor breeding efficiency, and elevated body temperature [10] have all been attributed to grazing endophyte-infected tall fescue. As horses are much better at dissipating heat than cattle, most classes of horses are more tolerant to fescue toxicity, though vasoconstriction has been documented in non-pregnant mares [11]. On the other hand, pregnant mares are more sensitive to ergovaline than cattle; mares grazing toxic tall fescue often go several weeks past their due date, resulting in large foals, dystocia, and in some cases foal or mare mortality [12]. These mares can also have thickened or retained placentas [13], further complicating the foaling process. Mares on toxic tall fescue often have limited or no milk production [14,15].

In recent years, novel endophyte tall fescue cultivars have been developed that contain beneficial *Epichloë* endophyte strains. These endophytes provide increased plant persistence and produce non-toxic alkaloids, which confer insect and nematode resistance, but do not cause livestock toxicity. Recent studies have determined that most novel endophyte tall fescue cultivars do not produce significant portions of ergot alkaloids, and mares grazing them are likely to foal normally [16]. However, some cultivars do still produce low levels of ergovaline, below threshold values for cattle, but high enough to potentially cause toxicity in last trimester broodmares [17,18].

The University of Kentucky Horse Pasture Evaluation Program began in 2005 in response to the outbreak of Mare Reproductive Loss Syndrome (MRLS) of 2001/2002. While it was eventually determined that the Eastern Tent Caterpillar was the causal agent for MRLS [19], this event made many horse farm managers acutely aware of the amount of toxic tall fescue in their pastures. The UK Horse Pasture Evaluation Program is a fee-based service offered by University of Kentucky Forage Extension to collect on-farm pasture data and make management recommendations. This program has experienced tremendous growth, in part due to the increasing concerns of tall fescue toxicity on broodmare farms. Here, we will present two case studies where tall fescue toxicity was evaluated on horse farms, and discussion on how ergovaline in total diet is calculated and used to make pasture recommendations.

## 2. Results

Two case studies will be presented below to showcase how data collected on farms has been used to make management recommendations. Both farms are commercial thoroughbred breeding operations in central Kentucky and have participated in the UK Horse Pasture Evaluation Program for several years. Farm names have been redacted to protect privacy.

### 2.1. Farm #1

Pastures in Farm #1 were sampled in the late summer of 2020. This farm had significant tall fescue composition, ranging from 23–73% and a farm average of 45%. Endophyte infection was high, averaging 84%, and ergovaline concentration in the fescue ranged from 288 to 977 ppb (parts per billion) across pastures (Table 1). As both ergovaline and tall fescue composition were high, there was minimal ergovaline dilution on this farm. Ergovaline in total diet was over the 200 ppb threshold for late term pregnant mares in 11 of the 15 fields. Although some were just over the threshold, others, such as paddock N, had ergovaline in total diet of 699 ppb, and therefore would be unsafe to hold pregnant mares during their last trimester. In contrast, paddock I showed an ergovaline in total diet of 181 ppb and therefore should be safe for grazing at the time of sampling. Paddock I may show higher ergovaline in the spring, as fescue composition was 32% and endophyte infection was 91%, and may require remediation or additional testing. Total reestablishment was recommended on several fields on this farm. 

### 2.2. Farm #2

Farm #2 had been sampled regularly for a number of years and had followed recommended toxic tall fescue mitigation strategies based on UK Horse Pasture Evaluation Program recommendations. Overall, tall fescue endophyte infection was high at 82% (Table 2), but no fields were of major concern due to two main factors: strong stands of other forages such as Kentucky bluegrass (*Poa pratensis* L.) and orchardgrass (*Dactylis glomerata* L.) which diluted ergovaline consumption, and healthy stands of novel endophyte tall fescue in several pastures. Pasture M1 had been monitored yearly as there was significant tall fescue composition, but ergovaline remained at moderate concentrations and there was a good stand of bluegrass. These combined factors, and the assumption that horses eat randomly in the pasture [20], meant that, in most years, ergovaline in total diet on M1 was low and mares could safely graze. If bluegrass composition declined or ergovaline increased, then this field may become a candidate for total reestablishment. In contrast, Pasture M3 had shown significant toxic tall fescue presence and other weeds issues in past years, therefore a total herbicide kill and reestablishment was recommended. M3 was replanted with a mixture of novel endophyte tall fescue, Kentucky bluegrass, and orchardgrass, and was monitored yearly to watch for toxic fescue encroachment. High endophyte and low ergovaline indicated the presence of a novel endophyte. By following pasture management recommendations, being willing to make changes when needed, and yearly pasture monitoring, this farm has significantly reduced the risk of tall fescue toxicity in broodmares. In the process, they have also reported that their newly established pastures showed improved foal growth rates and the additional forage production allowed hay harvests, thereby reducing purchased feed costs.

## 3. Discussion

The current literature does not pinpoint an exact threshold of ergovaline in total diet tolerated by pregnant mares, in part due to differences in study designs, feeding methods, pasture sampling, analysis, and reporting. Threshold values set by various extension services range from 0 to 300 ppb. At the University of Kentucky, 200 ppb is the generally accepted threshold value (Table 3), based on work that demonstrated palmar artery constriction around 200 ppb in mares [16]. Pastures testing in the 201–500 ppb range provide a risk when grazed by late term pregnant mares, but some of this risk can be mitigated if steps are taken to reduce ergovaline in total diet, such as mowing to remove seedheads or feeding hay. Pastures with more than 500 ppb in total diet are unlikely to be safe for mares in the last trimester of pregnancy, therefore the recommendation is to remove mares from these pastures.

**Figure 1:**
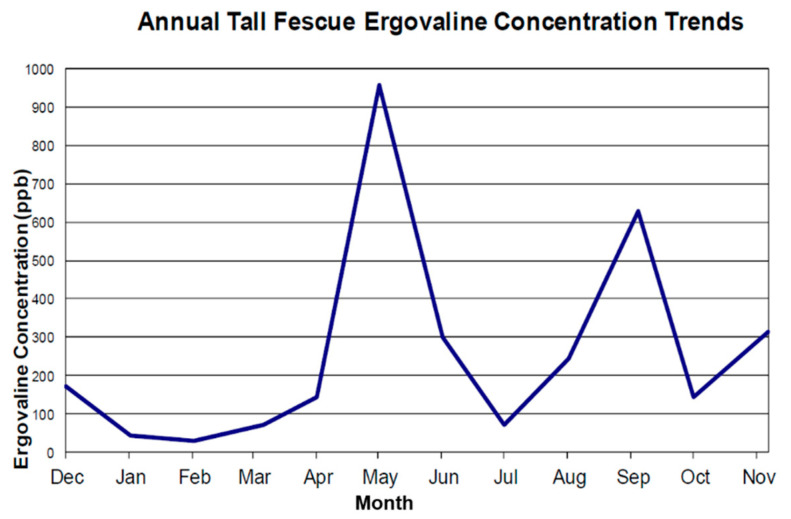
Season variation of ergovaline concentration based on monthly samples during one calendar year from a Kentucky pasture.

Following each evaluation, considerable time is spent with each horse farm owner/manager to educate them on the variations in ergovaline concentration in tall fescue. Common sources of variation include season, temperature, precipitation, and pasture management. Ergovaline concentrations are known to vary seasonally, and closely follow the cool season growth curve of the endophyte’s host, tall fescue. Figure 1 illustrates seasonal spikes in ergovaline concentration commonly seen in the spring and fall [21].

**Figure 2:**
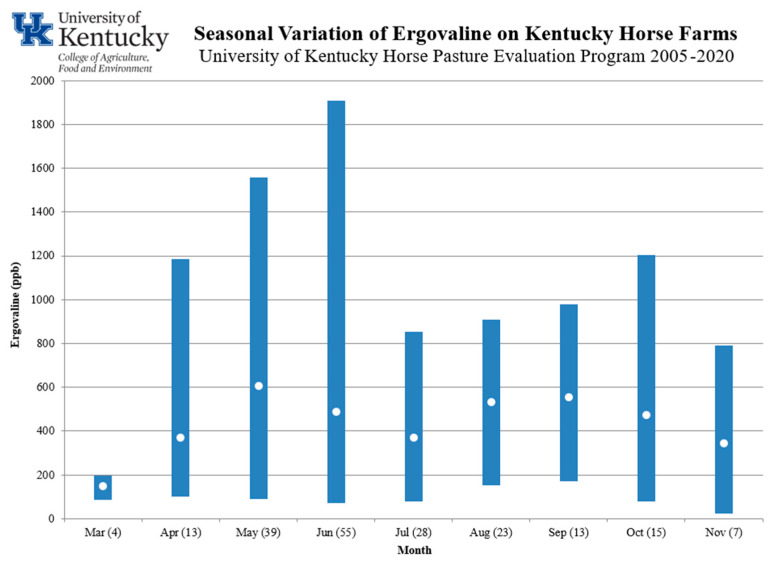
Range (bars) and average (dots) of ergovaline farm averages by month (number of samples) for all program participants since 2005.

Year to year variation is also significant and is not completely understood. Figure 2 provides a bar chart of the range in ergovaline concentration and averages across farms that have participated in this program since 2005. As in Figure 1, a bimodal curve is apparent for average ergovaline concentration, but the range in any given month is also extensive. Annual sampling of pastures suggests that those with higher-than-average ergovaline concentrations one year are likely to be higher than average in subsequent years [22]. Therefore, although ergovaline fluctuates from year to year, the fields with consistently higher ergovaline concentrations should be avoided.

Research from Dillard and colleagues [17] found that ergovaline is the most concentrated in the leaf sheath, which is mainly below 10cm. This finding is significant on horse farms that routinely graze or mow closer to the ground than on cattle operations. Horse pastures are typically kept around 15 cm, but small turn-out paddocks are often grazed much closer and frequently have higher ergovaline concentrations compared to larger pastures with taller grazing height.

Endophyte testing was once considered key in understanding and quantifying the toxicity potential in any given pasture. Early studies involving toxic tall fescue simply compared endophyte-infected to endophyte free pastures, such as Putnam [12]. Later studies compared high and low infected pastures to examine toxicity in livestock, such as Aiken, [23]. More advanced analyses, which include ergovaline or total ergot alkaloid quantification, provide a snapshot of toxicity potential in a pasture, but endophyte testing still provides a valid indicator of potential across a season. Endophyte percentage stays relatively unchanged from year to year, and high endophyte percentage suggests that, even if ergovaline or total ergot alkaloids are low at the time of testing, the potential is there for these to dramatically increase and become problematic under different conditions. The University of Kentucky Horse Pasture Evaluation program reports an average of 78% endophyte infection rate across all farms sampled in Kentucky 2005–2020. In addition, when combined with ergovaline analysis at peak times (spring and fall), the presence of a novel endophyte can be confirmed. Novel endophyte tall fescue pastures will have high endophyte percentages, but low ergovaline concentrations.

Average tall fescue presence in sampled horse pastures over the last 15 years is 18%, but the actual composition of tall fescue in individual pastures has ranged from 3 to 73%. Considering this wide range for tall fescue and the fact that ergovaline is not tested on many farms, a more relative risk scale can be used (Table 4). This scale is also useful when ergovaline was tested outside the normal months that late term pregnant mares are on pasture. For example, when tall fescue composition is less than 10% and there are good percentages of other forages, then a pasture would be considered a low risk for tall fescue toxicity. At higher levels of tall fescue, more radical approaches are necessary, unless the fescue is a novel endophyte variety proven by very low ergovaline concentrations.

Final management recommendations are made based on species composition and tall fescue sample results and the other factors that have been discussed. Recommendations may include seeding, spraying, complete renovation, mowing, fertilizing, hay feeding, or removing horses from a pasture. All results and recommendations are discussed with managers to help them find a solution that works for them and their operation.

## 4. Conclusions

Endophyte and ergovaline testing are key elements of understanding and reducing on-farm risk of tall fescue toxicosis in horses and other livestock. Pasture composition measurements are an additional indicator of toxicosis risk and should always be considered before making management changes. The University of Kentucky Horse Pasture Evaluation Program has found that recommendations based on this data and one-on-one discussions with farm owners/managers has led to improved pasture health, reduced tall fescue toxicity and better forage quality and quantity. Additionally, complete re-establishment has proven successful in mitigating tall fescue toxicity as well as other pasture challenges, such as warm season annual grasses, nimblewill (*Muhlenbergia schreberi* J.F. Gmel.), broadleaf weeds, and bare soil. Since 2005, the UK Horse Pasture Evaluation Program has completed 281 evaluations on over 170 farms in central Kentucky, representing over 67,000 total pasture acres. Recent improvements, including modified sampling methods and digital data collection, have streamlined the evaluation process, providing more consistent information with a rapid turnaround.

## 5. Materials and Methods

Pastures were sampled between April and November by trained individuals. From 2005 to 2019, visual estimation of a 0.65 × 0.65 m quadrat (made with 3.5 cm diameter PVC pipe), was conducted in 10–20 locations to determine species composition. Pastures less than 2 hectares had 10 sample locations; those 2–4 hectares had 15 sample locations and pastures greater than 4 hectares contained 20 sample locations. Pastures greater than 16 hectares were sub-divided and sampled accordingly. In 2020, pasture sampling was transitioned to the occupancy method as described by Vogel and Masters [24] and Payne [25]. Grids are 0.75 m × 0.75 m and contain 25 smaller squares, 15 cm × 15 cm each, made from metal cattle panels and cut to size with sharp edges ground off and painted for visibility. For each smaller square, the most dominant component was identified. Categories included tall fescue, Kentucky bluegrass, orchardgrass, white clover (*Trifolium repens* L.), broadleaf weeds, nimblewill, and bare soil (which includes warm season annual grasses).

A representative sample of tall fescue plant material was collected from each pasture. The plant material was harvested from 10 to 20 random locations, depending on the size of the pasture, to obtain a minimum total fresh sample weight of at least 300 g. Samples are cut 7–10 cm above the ground and included seedheads, if present. Material was placed on ice in a cooler, and transported to the University of Kentucky Veterinary Diagnostic Laboratory (Lexington, KY, USA. http://vdl.uky.edu/ (accessed on 24 September 2021)) for analysis. The lab flash froze samples in liquid nitrogen and utilized ultra-High Performance Liquid Chromatography with fluorescence detection to quantify ergovaline and its isomer, ergovalinine, as total ergovaline concentration. The full method is described in Lea et al. [26].

Twenty tall fescue tillers were collected throughout each pasture, individual tillers were cut at the soil surface, and placed in a cooler on ice for transport to the University of Kentucky Regulatory Services Lab following the procedure described by Vincelli [27]. Percentage of plants infected with an endophyte was determined using Agrinostics tiller test kit (Watkinsville, GA, USA).

For each pasture sampled, individual grid and total pasture species composition were provided, as well as ergovaline concentration (ppb) and endophyte (%) on a comprehensive datasheet. In addition, “ergovaline in total diet” was calculated using the following formula:

Ergovaline in total diet (ppb) = (%Tall Fescue/(% Tall Fescue + % Bluegrass + % Orchardgrass + % White Clover)) * ergovaline.

Management recommendations are based on all data collected and may include overseeding, chemical weed control, tall fescue mitigation or complete re-establishment (two rounds of glyphosate is recommended in late summer and early fall, followed by a fall seeding of perennial cool-season grasses). Seeding mixtures typically include Kentucky bluegrass and orchardgrass, and often a novel endophyte tall fescue.

## Figures and Tables

**Table 1:** Data from a representative thoroughbred breeding farm in central Kentucky (Farm #1) showing field acreage, species composition, endophyte infection, ergovaline concentration, and calculated ergovaline in total diet.

Field	TF ^1^	BG	OG	WC	WD	NW	BS	Endophyte	Ergovaline	Ergovaline in Total Diet
	------------------------------------------%------------------------------------------								------------ppb------------	
Pasture 1	23	27	23	0	8	1	19	80	552	178
Pasture 2	33	30	21	0	7	1	7	83	485	189
Pasture 3	46	24	8	0	0	2	15	85	547	321
Pasture 4	73	13	8	0	0	0	6	81	406	314
Pasture 5	57	27	8	0	1	2	4	95	454	283
Pasture 6	38	39	7	0	2	1	13	83	634	287
Pasture 8	43	18	16	0	5	0	15	86	552	306
Paddock E	34	37	20	0	0	0	9	88	542	203
Paddock H	40	9	30	0	8	5	4	94	609	311
Paddock I	32	40	10	0	6	1	10	91	465	181
Paddock J	48	18	20	0	0	1	13	76	288	161
Paddock K	37	17	28	0	0	1	18	95	545	247
Paddock N	59	11	13	0	0	0	14	79	977	699
Paddock T	46	2	14	0	0	2	36	76	415	311
Paddock U	60	2	31	0	0	0	7	67	366	237
Average	45	21	17	0	3	1	13	84	522	282

^1^ TF = Tall Fescue, BG = Kentucky Bluegrass, OG = Orchardgrass, WC = White Clover, WD = Weeds, NW = Nimblewill, and BS = Bare Soil and Warm Season Annual Grasses.

**Table 2:** Data from a thoroughbred breeding farm in central Kentucky (Farm #2) following toxic tall fescue mitigation strategies showing species composition, endophyte infection, ergovaline concentration, and calculated ergovaline in total diet.

Field	TF ^1^	BG	OG	WC	WD	NW	BS	Endophyte	Ergovaline	Ergovaline in Total Diet
	------------------------------------------%------------------------------------------								------------ppb------------	
Pasture M1	22	25	10	1	3	25	4	92	422	162
Pasture M3	44	10	4	1	2	10	17	73	<100 ^2^	<100
Field 9	11	19	40	7	2	14	1	90	247	35
Field 10	7	15	20	13	6	29	3	67	289	39
Paddock IP1	15	0	7	3	4	3	58	95	<100	<100
Paddock IP2	67	8	4	0	2	6	12	80	<100	<100
New Paddock	20	7	3	0	2	30	17	90	<100	<100
Field 01	28	11	10	1	12	26	4	75	<100	<100
LC Field 1	13	18	21	23	2	20	1	75	171	31
LC Field 2	4	12	16	6	4	38	17	86	165	18
Farm Average	23	12	13	5	4	20	13	82	259	57

^1^ TF = Tall Fescue, BG = Kentucky Bluegrass, OG = Orchardgrass, WC = White Clover, WD = Weeds, NW = Nimblewill, and BS = Bare Soil and Warm Season Annual Grasses. ^2^ Lower quantification limit is 100 ppb for this method.

**Table 3:** Risk level for late term pregnant mares based on ergovaline concentration in total diet.

Ergovaline in Total Diet	Recommendation for Late-Term Mares
<200 ppb	Low risk—monitor for seasonal fluctuations
201–500 ppb	Risk—take steps to mitigate tall fescue in pasture
>500 ppb	High risk—remove mares from pasture in last 60 days of pregnancy

**Table 4:** Risk level to late term pregnant mares based on percent tall fescue composition and management recommendations based on the risk level.

Tall Fescue	Risk Level	Management Recommendation
<10%	Very small risk to late term mares.	The only risk would be during severe stress periods (e.g.,—in a hot, dry summer) when the tall fescue may be growing and the KY bluegrass is dormant or when other desirable species are not present.
10–25%	Risk to late term mares is small, but safe pregnancy not guaranteed.	If the last 60–90 days of pregnancy occur in late March/early April or late November/early December, then watch mares carefully. Suppressing tall fescue with herbicide (imazapic) can be considered.
25–50%	Risk to late term mares is significant, especially during stress periods.	Suppressing tall fescue with herbicide (imazapic) can be considered if grazing late term mares in this pasture. Overseed following spraying, but wait for at least two months residue will inhibit the growth of new seedlings.
50–75%	Risk to late term mares is high.	Do not graze pregnant mares during the last 60–90 days of pregnancy. Herbicides used to suppress fescue may result in bare ground and weed growth. Complete reestablishment is recommended if grazing late term mares.
75–100%	Risk to late term mares is very high.	Do not graze pregnant mares during the last 60–90 days of pregnancy.

## Data Availability

Not applicable.
